# Intrathecal Fluorescein in the Surgical Management of Spontaneous Cerebrospinal Fluid Leaks: A Case Report

**DOI:** 10.7759/cureus.103045

**Published:** 2026-02-05

**Authors:** Sara Costa, Simão Bessa, João Almeida, Telma Feliciano, João Lino

**Affiliations:** 1 Department of Otorhinolaryngology - Head and Neck Surgery, Unidade Local de Saúde de Santo António, Porto, PRT; 2 Department of Otorhinolaryngology - Head and Neck Surgery, Unidade Local de Saúde do Tâmega e Sousa, Porto, PRT

**Keywords:** endoscopic endonasal repair, intrathecal fluorescein, nasoseptal flap, skull base defect, spontaneous csf leak

## Abstract

Spontaneous cerebrospinal fluid (CSF) rhinorrhea can be difficult to localize because skull base defects may be small, multifocal, or inconspicuous on imaging. Intrathecal fluorescein has been used as an adjunct to improve intraoperative identification of CSF leaks during endoscopic repair. We report the case of a 27-year-old obese woman with a two-week history of right-sided clear rhinorrhea and orthostatic headache. Biochemical analysis of the nasal discharge was consistent with CSF. Computed tomography suggested skull base vulnerability but did not clearly identify the leak site. Low-dose, diluted intrathecal fluorescein was administered via lumbar puncture with slow injection prior to endoscopic endonasal surgery. Intraoperatively, vivid fluorescence precisely localized a dural defect at the right posterior ethmoidal roof, enabling targeted repair with a vascularized nasoseptal flap and adjunctive sealants. No fluorescein-related adverse effects occurred. While the repaired side showed no persistence, the patient developed new contralateral rhinorrhea on postoperative day one, requiring multidisciplinary reassessment and subsequent neurosurgical repair via craniotomy. Intrathecal fluorescein can be a valuable and safe adjunct (when used in low doses with appropriate dilution and slow administration) to localize radiologically occult spontaneous CSF leaks and to confirm repair integrity intraoperatively, though spontaneous leaks may be complex and warrant close surveillance and multidisciplinary management.

## Introduction

Cerebrospinal fluid (CSF) leak is a potentially serious condition owing to the risk of meningitis or neurological injury [1]. Endoscopic endonasal surgery is currently the treatment of choice, as it provides direct, minimally invasive access to the skull base [1,2]. Prompt diagnosis and precise localization of the skull base defect are essential to ensure a successful surgical repair. However, this is particularly challenging in cases of spontaneous CSF leaks, in which defects are often small, multifocal, or radiologically inconspicuous [3]. In this setting, intrathecal fluorescein has been described as a valuable adjunct for enhancing intraoperative identification and localization of CSF leaks during endoscopic endonasal repair [4-7].

## Case presentation

We report the case of a 27-year-old obese woman who presented to the emergency department with a two-week history of right-sided clear rhinorrhea associated with orthostatic headache. There was no history of prior head trauma or surgery. Biochemical analysis of the nasal discharge revealed elevated glucose and protein levels, compatible with CSF. Computed tomography of the paranasal sinuses revealed a low ethmoid roof and thinning of the cribriform plate, but failed to accurately identify the site of the leak.

**Figure 1:**
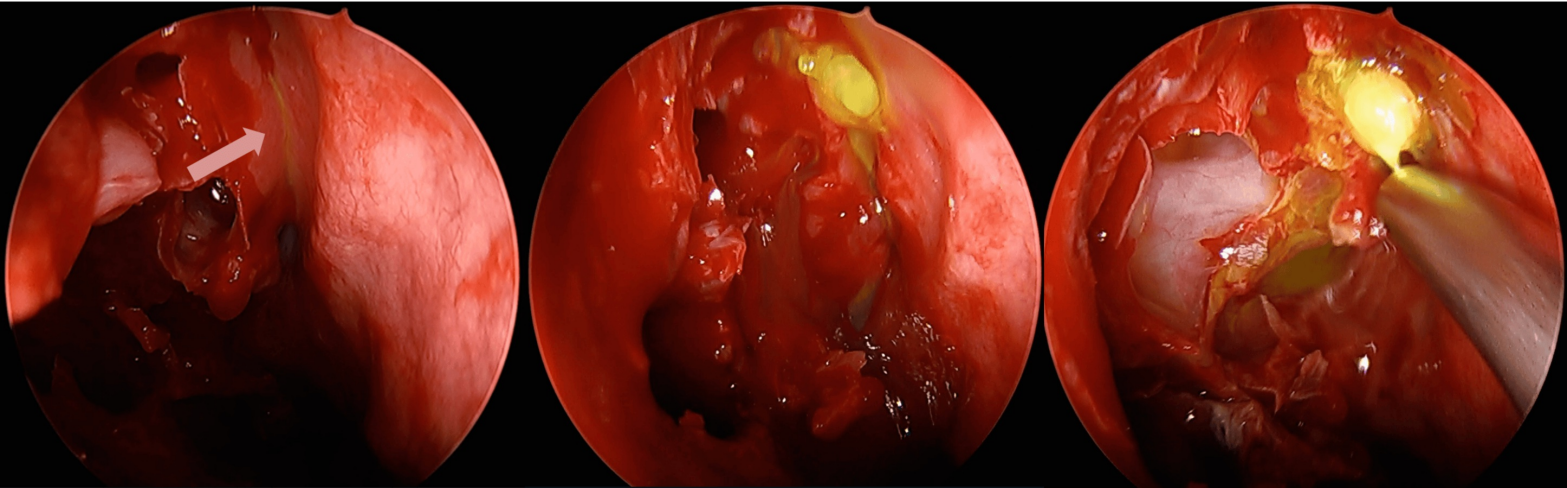
Endoscopic endonasal view demonstrating green fluorescent CSF leakage at the level of the right posterior ethmoidal roof following intrathecal fluorescein administration

Given the clinical suspicion of a spontaneous CSF fistula, the patient was scheduled for endoscopic endonasal repair, preceded by adjunctive intrathecal fluorescein administration. Under sterile conditions and following lumbar puncture, a low dose of 5% fluorescein diluted in autologous CSF was administered intrathecally using a slow and controlled injection. Intraoperatively, vivid green fluorescent leakage was observed at the level of the right posterior ethmoidal roof, allowing precise localization of the dural defect (Figure 1).

**Figure 2:**
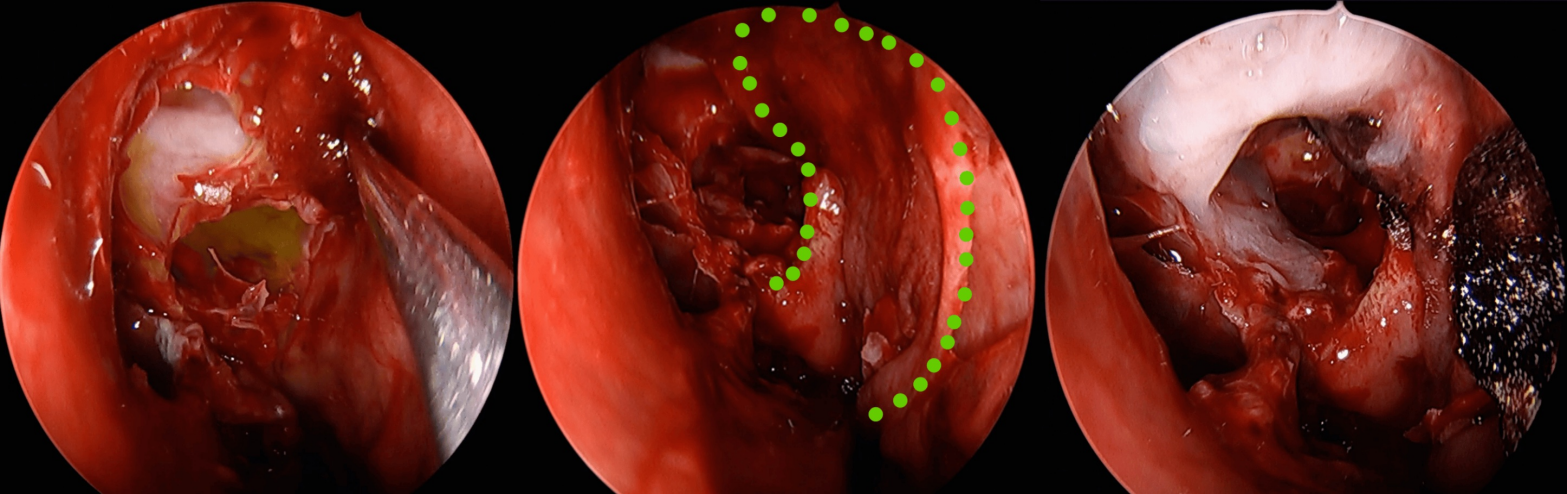
Endoscopic endonasal view after skull base reconstruction with a vascularized nasoseptal flap, showing absence of residual fluorescence and no evidence of persistent CSF leak

Reconstruction was performed using a vascularized nasoseptal flap pedicled on the posterior nasal septal artery, reinforced with oxidized regenerated cellulose and fibrin glue (Figure 2). After the repair, no residual fluorescence or active leakage was observed endoscopically.

No neurological symptoms, seizures, or systemic adverse effects related to fluorescein administration were observed. The postoperative progress revealed no ipsilateral recurrence. However, on the first postoperative day, the patient developed new-onset contralateral rhinorrhea. The case was discussed in a multidisciplinary setting, and the patient underwent re-operation by the Neurosurgery team, using an external approach via craniotomy and a pericranial flap for repair.

## Discussion

CSF leaks most commonly arise from craniofacial trauma, followed by iatrogenic causes, predominantly after sinonasal procedures [1,2]. Spontaneous CSF leaks are less frequent and are often associated with elevated intracranial pressure or idiopathic intracranial hypertension [1,3]. In these cases, skull base defects are frequently subtle and may evade detection on conventional imaging modalities, rendering precise localization of the leakage site challenging in a subset of patients.

Intrathecal fluorescein enhances contrast between CSF and surrounding tissues, facilitating precise localization of the defect and enabling real-time verification of repair integrity [4-7]. This intraoperative confirmation may reduce recurrence rates and the need for revision surgery. In the present case, fluorescein proved particularly useful given the inconclusive imaging findings and the suspected spontaneous etiology.

Historically, concerns regarding neurotoxicity limited the widespread use of intrathecal fluorescein. Reported complications, including seizures and neurological deficits, were primarily associated with excessive doses, inadequate dilution, or rapid administration. More recent evidence supports the safety of intrathecal fluorescein when low doses are used, diluted appropriately, injected slowly, and accompanied by close perioperative monitoring [5,8].

## Conclusions

This case underscores the clinical value of intrathecal fluorescein as an adjunct in the endoscopic repair of spontaneous CSF leaks. Careful patient selection and strict adherence to established safety protocols (low doses, appropriate dilution, slow injection, and close monitoring) are essential to optimize outcomes while minimizing potential risks. Although this approach was effective in managing the initial CSF leak site, the occurrence of new-onset contralateral leakage postoperatively illustrates the inherent complexity of these cases and emphasizes the need for meticulous clinical surveillance and a multidisciplinary management strategy.

## References

[1] Hall WA, Schaurich CG, Rout P (2025). Cerebrospinal fluid leak. StatPearls [Internet].

[2] McMains KC, Gross CW, Kountakis SE (2004). Endoscopic management of cerebrospinal fluid rhinorrhea. Laryngoscope.

[3] Martínez-Capoccioni G, Serramito-García R, Martín-Bailón M (2017). Spontaneous cerebrospinal fluid leaks in the anterior skull base secondary to idiopathic intracranial hypertension. Eur Arch Otorhinolaryngol.

[4] Jolly K, Gupta KK, Muzaffar J, Ahmed SK (2022). The efficacy and safety of intrathecal fluorescein in endoscopic cerebrospinal fluid leak repair - a systematic review. Auris Nasus Larynx.

[5] Mackie H, Eide JG, Yassin-Kassab A (2025). Rapid intrathecal fluorescein injection during cerebrospinal fluid leak repair is safe and effective. Laryngoscope.

[6] Tabaee A, Placantonakis DG, Schwartz TH, Anand VK (2007). Intrathecal fluorescein in endoscopic skull base surgery. Otolaryngol Head Neck Surg.

[7] Aljubran HJ, Alzamil MF, Alnasser AA, Bashammakh MM, Alfaleh MA, Almomen A (2025). The clinical applications of intrathecal fluorescein in cerebrospinal fluid leak repair. Radiol Case Rep.

[8] Rodríguez-Navarro MÁ, Díaz-Alejo C, Padilla-Del Rey ML, Alcaraz AB, González-Pérez P, Benítez M (2017). Safe intrathecal fluorescein use for identification of cerebrospinal fluid leaks: case-report and perioperative algorithm description. Rev Esp Anestesiol Reanim.

